# Test-Retest Reliability of fMRI During Nonverbal Semantic Decisions in Moderate-Severe Nonfluent Aphasia Patients

**DOI:** 10.1155/2004/974094

**Published:** 2005-01-27

**Authors:** Jacquie Kurland, Margaret A. Naeser, Errol H. Baker, Karl Doron, Paula I. Martin, Heidi E. Seekins, Andrew Bogdan, Perry Renshaw, Deborah Yurgelun-Todd

**Affiliations:** ^1^Harold Goodglass Boston University Aphasia Research CenterDepartment of NeurologyBoston University School of Medicine and the Veterans Affairs Boston Healthcare SystemUSA; ^2^University of Colorado at BoulderDepartments of SpeechLanguage and Hearing Sciences and NeuroscienceUSA; ^3^Brain Imaging CenterMcLean HospitalBelmontMA and Harvard Medical SchoolUSA

**Keywords:** nonfluent aphasia, fMRI reliability, nonverbal semantic decisions

## Abstract

Cortical reorganization in poststroke aphasia is not well understood. Few studies have investigated neural mechanisms underlying language recovery in severe aphasia patients, who are typically viewed as having a poor prognosis for language recovery. Although test-retest reliability is routinely demonstrated during collection of language data in single-subject aphasia research, this is rarely examined in fMRI studies investigating the underlying neural mechanisms in aphasia recovery.

The purpose of this study was to acquire fMRI test-retest data examining semantic decisions both within and between two aphasia patients. Functional MRI was utilized to image individuals with chronic, moderate-severe nonfluent aphasia during nonverbal, yes/no button-box semantic judgments of iconic sentences presented in the Computer-assisted Visual Communication (C-ViC) program. We investigated the critical issue of intra-subject reliability by exploring similarities and differences in regions of activation during participants' performance of identical tasks twice on the same day. Each participant demonstrated high intra-subject reliability, with response decrements typical of task familiarity. Differences between participants included greater left hemisphere perilesional activation in the individual with better response to C-ViC training. This study provides fMRI reliability in chronic nonfluent aphasia, and adds to evidence supporting differences in individual cortical reorganization in aphasia recovery.

## 1. Introduction

Cortical reorganization underlying poststroke language recovery, while not well understood, is now being actively studied with functional neuroimaging. Some studies link recovery with greater activation of right hemisphere (RH) language homologues [7,10,39]. These findings, however, have been challenged by studies suggesting that abnormal and/or over-activation of RH structures during language tasks may be, in part, a maladaptive process [1,29,30,35]. Other studies suggest that activation of residual left hemisphere (LH) perilesional areas may be critical to better, or more efficient, language recovery [5,14,15,30,40]. Some reasons underlying the different conclusions offered by these studies include heterogeneity of subjects, task selection, and differences in methodologies used to acquire data [6].

Recently, it has been suggested that important insights can be gained via the single-subject approach to studying aphasia recovery with functional imaging [30]. A well-established methodological issue in

ISSN 0953-4180/04/$17.00 © 2004 – IOS Press and the authors. All rights reserved single-subject research is the need for test-retest reliability [20]. Test-retest reliability provides a measure of stability, repeatability, and consistency over time. Although test-retest reliability is routinely performed during collection of behavioral data, it is still fairly uncommon among published studies investigating the underlying neural mechanisms supporting those behaviors. This oversight may be due to the relatively high cost and low availability of functional neuroimaging time. Nonetheless, the issue of test-retest reliability in functional neuroimaging research remains important.

Previous functional neuroimaging and lesion studies investigating semantic processing in healthy normal controls and aphasia patients have suggested a broad network of regions to be necessary and/or sufficient in the performance of lexical-semantic tasks [10,11, 13,18,19,22–24,30,31,39]. This semantic network includes, but is not limited to, the following areas: superior, middle and inferior temporal gyri (BA 22, 21, 20), fusiform gyrus (T4 and BA 18, 19, 37), temporal pole (BA 38), SMA (BA 6), angular gyrus (BA 39), supramarginal gyrus (BA 40), prefrontal areas (BA 8, 9, 10, 46, 47), and posterior inferior frontal gyrus (BA 44, 45). These studies examined a wide variety of healthy and language impaired subjects (of varying type and severity of aphasia) during performance of variable receptive and expressive semantic tasks. No two studies suggest the exact same necessary and/or sufficient regions, but this is not surprising given differences in subjects, tasks and control conditions that differentially emphasize various aspects of the complex phenomenon of lexical-semantic processing.

We have utilized functional imaging to investigate the neural networks supporting language recovery in aphasia. The present study is part of a larger investigation into semantic decision-making in patients with chronic, severe nonfluent aphasia. These patients have been treated with the iconic, nonverbal Computer-assisted Visual Communication (C-ViC) program [16, 17]. To our knowledge, no other functional imaging studies with aphasia patients have reported results post-treatment with an alternative nonverbal communication system. Moreover, none of the studies investigating semantic processing in aphasia patients have reported test-retest reliability.

This study addresses the issue of test-retest reliability in two individuals with chronic, moderate-to-severe nonfluent aphasia during acquisition of blood oxygenation level dependent (BOLD) functional magnetic resonance imaging (fMRI). During fMRI, they performed a nonverbal, yes/no, button-box semantic judgment task on C-ViC icon "sentences". We investigated the critical issue of intrasubject reliability by exploring similarities and differences in regions of activation during each participant's performance of two identical tasks on the same day. We also explored hemispheric lateralization and localization associated with different degrees of aphasia recovery in these two patients.

We hypothesized the following: 1) Patients with moderate-to-severe nonfluent aphasia can provide reliable fMRI data during performance of language tasks in the scanner; 2) During fMRI, these patients will activate RH regions homologous to LH regions previously associated with lexical-semantic functions. These patients may also activate undamaged LH regions as well as perilesional regions; 3) The patient with "best response" to C-ViC training [28] will demonstrate better performance (accuracy and response times), and will have a different pattern of fMRI activation than the patient with "moderate response", including more LH activity in the patient with "best response".

## 2. Methods

### 2.1. Participants

Each participant suffered a single left hemisphere stroke (Figs 1 and 2). Participant 1 (P1) is a right-handed, 69-year old man, former carpenter, 11 years poststroke onset, with severe nonfluent speech. Structural MRI scan revealed that both Broca's and Wernicke's cortical areas were spared. This patient primarily had a subcortical lesion centered over the putamen with lesion extension into two white matter areas near ventricle, compatible with nonfluent speech (arrows on MRI scan in Fig. 1): 1) medial subcallosal fasciculus (MScF), deep to Broca's area, adjacent to the left (L) frontal horn (affecting pathways from SMA and cingulate gyrus BA 24 to head of caudate); and 2) middle 1/3 periventricular white matter (M 1/3 PVWM), located deep to sensorimotor cortex, adjacent to the L body of lateral ventricle (affecting sensori-motor pathways deep to mouth, inter- and intra-hemispheric pathways including in part, limbic and motor thalamo-cortical pathways) [27]. Some cortical lesion was present in the supramarginal gyrus and part of the angular gyrus.

P2 is a right-handed, 59-year old man, former construction engineer, 10 years poststroke onset, with moderate-severe nonfluent speech. Structural MRI scan showed cortical lesion in all of Broca's area and portion of Wernicke's area. Subcortical white mat-

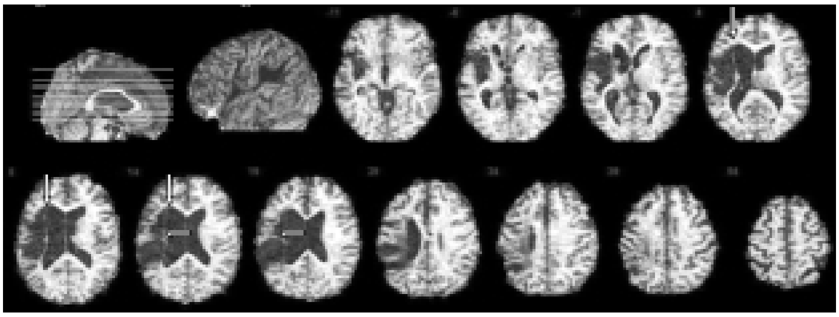

Fig. 1. Structural T1-weighted, three-dimensional spoiled gradient echo (3D SPGR) MRI scan for P1 (severe nonfluent, "moderate" response to C-ViC training). Both Broca's and Wernicke's cortical areas were largely spared. Subcortical lesion was centered over the putamen with lesion extension into two white matter areas near ventricle, compatible with nonfluent speech: 1) medial subcallosal fasciculus (MScF), deep to Broca's area, adjacent to the left (L) frontal horn (vertical arrows); and 2) middle 1/3 periventricular white matter (M 1/3 PVWM), located deep to sensorimotor cortex, adjacent to the L body of lateral ventricle (horizontal arrows). Some cortical lesion was present in the supramarginal gyrus and part of the angular gyrus.

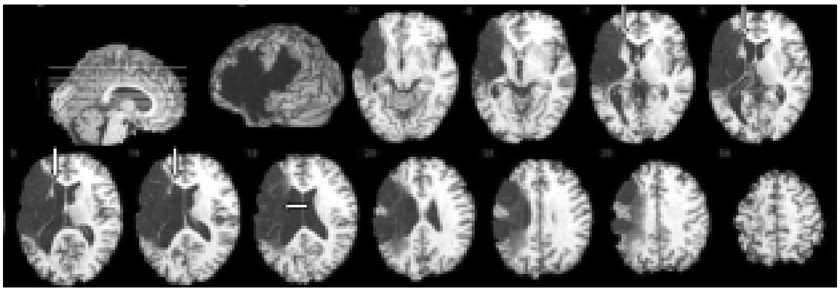

Fig. 2. Structural T1-weighted, 3D SPGR MRI scan for P2 (moderate-severe nonfluent, "best" response to C-ViC training). Cortical lesion was present in all of Broca's area and portions of Wernicke's area. Subcortical white matter lesion was present in both white matter areas near ventricle, compatible with nonfluent speech: 1) MScF (vertical arrows); and 2) M 1/3 PVWM (horizontal arrows). Cortical lesion was present in lower sensorimotor cortex (mouth region) with sparing in the upper regions; lesion was also present in the supramarginal gyrus and part of the angular gyrus.

ter lesion was present in both white matter areas near ventricle, compatible with nonfluent speech (arrows on MRI scan in Fig. 2): 1) MScF; and 2) M 1/3 PVWM. Cortical lesion was present in lower sensorimotor cortex (mouth region) with sparing in the upper regions; lesion was also present in supramarginal gyrus and part of the angular gyrus.

Longitudinal scores in auditory comprehension, repetition and naming on the Boston Diagnostic Aphasia Exam (BDAE) [12] demonstrate chronic nonfluent aphasia (Table 1). P1 at 6.5 Yr. poststroke had severe nonfluent speech (0-1 word phrase length). When P2 entered C-ViC training at 2 Yr. poststroke, he had severe nonfluent speech (0–1 word phrase length). By 10 Yr. poststroke, P2 had moderate nonfluent speech (2–3 word phrase length). Neither patient was classified as having global aphasia because each had relatively preserved auditory comprehension (70th percentile). The study was approved by the Institutional Review Boards at all hospitals where the authors are affiliated, and signed informed consent was obtained.

### 2.2. *fMRI experimental design*

Functional MRI measurements were collected while participants made yes/no button-box semantic decisions regarding appropriateness of icon "sentences" presented in Computer-assisted Visual Communication (C-ViC) [35]. C-ViC is an icon-based alternative communication system designed for patients with severe aphasia including limited oral, gestural, or written expressive output. Both participants had received at least 9 months of training in the C-ViC program with one of the authors (EB) within four years of undergoing this fMRI experiment. Both attended several weeks of C-ViC refresher training with EB during the month prior to this fMRI study. In response to previous C-ViC training, P1 had been classified as having "moderate response", whereas P2 had been classified as having

Table 1
Boston Diagnostic Aphasia Exam (BDAE) test scores for participants

| Patient data | | | Fluency | Auditory comprehension | | | | Repetition | Naming |
|---|---|---|---|---|---|---|---|---|---|
| Patient | MPO when tested | Date when tested | Max. No. words per phrase | AC: Mean of 3 %iles | Word discrim | Commands | Complex ideational material | Single word repetition | Visual confr'n. naming |
| | Maximum score: | | 7 | | 72 | 15 | 12 | 10 | 114 |
| 1/ severe | 4.5 Yr. | 4/97[a] | 0-1 | 72.3 | 66 | 14 | 6 | – | – |
| nonfluent speech | 6.5 Yr. | 2/99[b] | 0-1 | 69.3 | 68 | 11 | 7 | 1 | 8 |
| 2/ mod-severe | 10 Mo. | 7/94[c] | 0-1 | 38.3 | 46 | 9 | 3 | 8 | 28 |
| nonfluent | 4 Yr. | 3/97[d] | 2 | 62.1 | 62.5 | 8 | 9 | 8 | 59 |
| speech | 10 Yr. | 5/03[e] | 2-3 | 74.3 | 65 | 13 | 9 | 9 | (12/20) |

[a]2 Yr. post-C-ViC training
[b]4.5 Yr. prior to fMRI study
[c]immediately pre-C-ViC training
[d]2 Yr. post-C-ViC training
[e]6 Mo. prior to fMRI study

"best response" [28]. While both individuals could utilize C-ViC to respond to questions posed by others, only P2 used C-ViC to initiate communication, and did so generally with better syntactic and semantic ability than did P1.

The C-ViC subject-verb-object icon "sentences" were either acceptable (e.g., "woman cut pizza") or unacceptable due to semantic unrelatedness (SUR) of the verb and object (e.g., "man build cheese"). See Fig. 3.

During fMRI sessions, participants viewed two runs of alternating blocks of rest (passively viewing "non-nameable" black and white patterns, 7.5 sec. per pattern) and the semantic decision task (7.5 sec. per icon sentence). Patients had been trained to press the left button to accept the icon sentence (if it "makes sense"), and the right button to reject it. Each run lasted 2.5 minutes, and each block of rest or semantic decision task lasted 30 seconds and consisted of four 7.5-second trials. After approximately 25 minutes of other MR imaging, and while the participant was still in the same position in the MRI scanner, the identical session with two runs of S-V-O sentences judged earlier, was repeated for test-retest fMRI reliability.

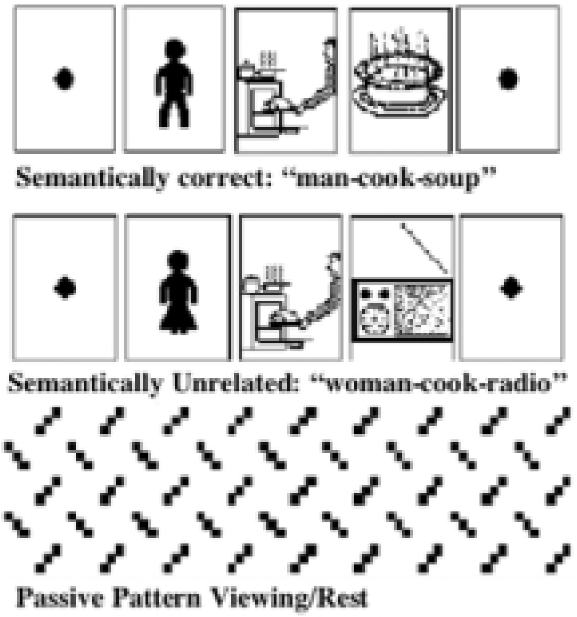

Fig. 3. C-ViC subject-verb-object (S-V-O) icon "sentences" were judged to be acceptable (e.g., "man cook soup") or they were judged to be unacceptable, due to semantic unrelatedness (SUR) of the verb and object (e.g., "woman cook radio"). During the passive pattern viewing/rest condition, one of six different black and white "non-nameable" patterns was viewed.

### 2.3. Data acquisition

Functional MRI images were acquired using a 1.5 T GE Signa scanner. Scout images were acquired in the sagittal plane in order to define the anterior commissure-posterior commissure (AC-PC) line. Functional images were acquired in the same plane (parallel to AC-PC) using a T2*-weighted gradient echo, EPI sequence with TR = 3000 ms, TE = 40 ms, FOV = 24 × 24 cm, 64 × 64 matrix size with an in-plane resolution of 3.75 mm and 30 slices of 5 mm thickness with no gap. A high-resolution three-dimensional spoiled gradient echo (3D SPGR) image was acquired at the end of the scanning session, TR = 35 ms, TE = 5 ms, FOV = 24 × 24 cm, 256 × 256 matrix size with an in-plane resolution of 0.94 mm and 124 slices of 1.5 mm thickness with no gap.

During each of the two, 2.5-minute fMRI runs, each condition lasted 30 seconds (4 stimuli at 7.5 seconds each), during which 10 scans were acquired. The resting (pattern) condition alternated with the yes/no button-box task condition, for a total of 50 volumes (30 pattern, 20 task for each run). After less than half an hour of performing other tasks in the scanner, with rest between tasks, these two 2.5-minute fMRI runs (TEST session) were repeated (RETEST session).

### *2.4. Data analysis*

Data were analyzed with MatLab 6.5 (MathWorks, Natick, MA) and SPM2 (Wellcome Department of Cognitive Neurology, London, UK) on a Dell Workstation Precision 360. Functional runs begin with 12 s, 4 images, of dummy scans to establish longitudinal magnetization. Images were realigned using the first (post-dummy) functional image as a reference. The mean realigned EPI image was coregistered to the 3D SPGR using mutual coregistration information with these orientation shifts applied to the realigned EPI time series. The 3D SPGR was normalized to the MNI T1 template and resampled to 2 $\times$ 2 $\times$ 2 mm matrix size. These warping parameters were then applied to the EPI time series. Functional data were smoothed with a 6 mm$^3$ FWHM isotropic Gaussian kernel. Each voxel was regressed against a box-car reference waveform convolved with a canonical hemodynamic response function and subsequent T-tests were performed [uncorrected $p < 0.001$; corrected Family Wise Error (FWE) $p < 0.05$] to determine task-related functional activation patterns contrasting semantic decisions with pattern viewing/rest. The FWE adjustment protects against family-wise false positives using a Gaussian field correction for spatially extended data that is analogous to the Bonferroni correction for discrete data. This procedure controls the FWE rate at or below alpha (0.05), which represents the chance of one or more false positives anywhere (not limited to supra-threshold voxels). Significantly activated voxels were transformed from MNI space to the standard stereotaxic space of Talairach and Tournoux [38] using MEDx 3.42 medical imaging processing software [3]. Graphic imaging was performed using MRIcro software [34].

## 3. Results

### *3.1. Behavioral results*

Figure 4 reports accuracy and response times (RTs, for accurate responses only) for each participant. Percent accuracy was stable across test-retest in each participant. Errors were not identical for either participant across sessions. P1 (“moderate response” to C-ViC training), correctly judged 12/16 (75%) icon sentences in the first session (TEST) and also 12/16 (75%) in the second session (RETEST). His errors (3/4 each session) predominantly consisted of accepting semantically unrelated icon sentences, i.e., judging them to be correct. For example, he judged the sentence, “man build cheese” to be semantically acceptable.

P2 (“best response” to C-ViC training), correctly identified 12/14 (86%) during the TEST session and 13/15 (87%) during the RETEST session. Although 16 stimuli were presented in each session (8 per run), some response data are missing due to excessively long reaction time (>7500 msec). In addition, not all fMRI data could be analyzed for P2, due to head motion artifact (>0.5 mm in any direction). Among the data that could be analyzed during the TEST session, he scored 5/7 (71%); during the RETEST session he also scored 5/7 (71%). Errors were mixed, i.e., both judging semantically acceptable sentences to be inaccurate, and accepting SUR sentences.

Paired t-tests between each participant’s own TEST and RETEST scores showed each subject was faster on RETEST (P1: mean difference = 407 msec, $t = 2.08$, $df = 11$, $p = 0.062$; P2: mean difference = 1480 msec, $t = 3.68$, $df = 4$, $p = 0.021$). See Fig. 4. There was no significant difference between the RTs of the two participants.

### *3.2. Functional MRI results*

Figure 5 shows activated regions superimposed on each participant’s reconstructed images during TEST and RETEST. Significant activation is shown at $p < 0.001$ uncorrected, for display purposes. Table 2 reports selected regions showing significantly higher BOLD activations during semantic decisions than during passive viewing/rest. These regions were selected from previously published functional neuroimaging studies investigating semantic processing, as reviewed above. Significant clusters in Table 2 are corrected for multiple comparisons using $p < 0.05$ Family-Wise Error correction. Some clusters demonstrated significance at the less conservative, $p < 0.05$ False Discovery Rate, especially during RETEST, and are noted as such (by asterisk).

In general, regions which were strongly activated during TEST were also activated during RETEST, but with less spatial extent and intensity of activation (lower $p$ levels) on RETEST. Normal subjects have also

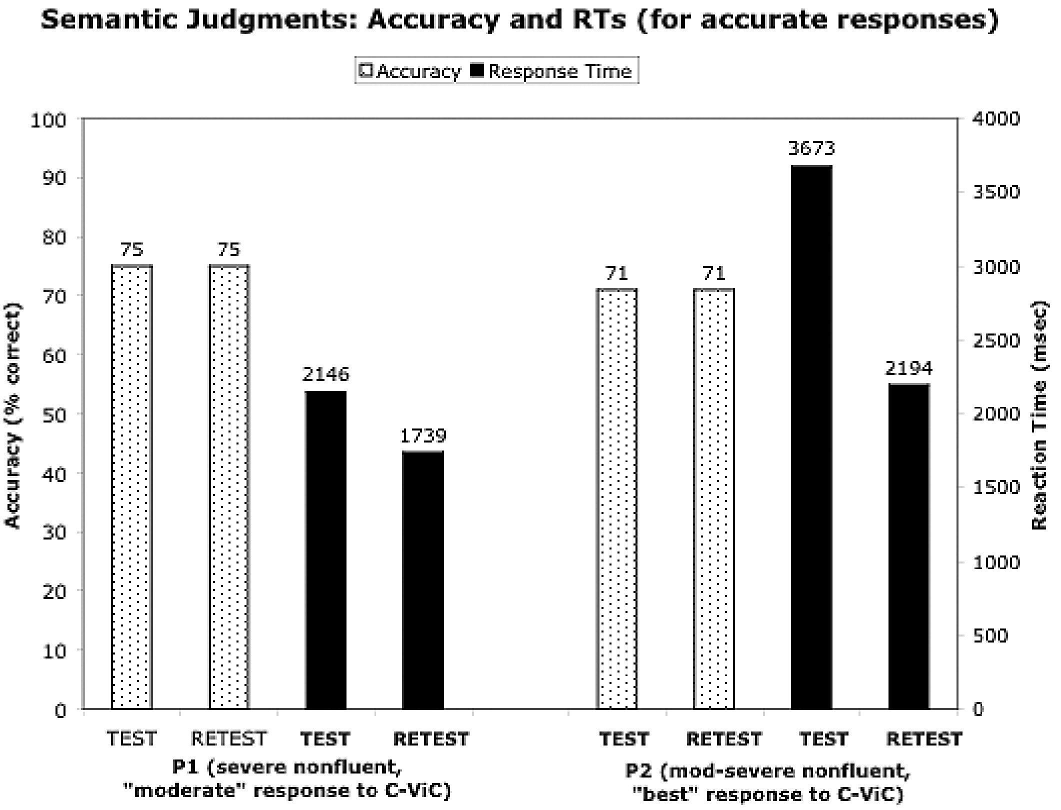

Fig. 4. Accuracy and response times (for accurate responses) for P1 and P2 during TEST and RETEST. Both participants showed no change in accuracy between TEST and RETEST runs, although errors were not identical between sessions. Both decreased RTs: P1 marginally significant ($t = 2.08$, $df = 11$, $p = 0.062$, mean difference $=$ 407 msec); P2 significant decrease in RT ($t = 3.68$, $df = 4$, $p = 0.021$, mean difference $=$ 1480 msec).

demonstrated this phenomenon of ‘repetition suppression’, i.e., decreased neuronal activation on delayed, identical tasks [4]. Results for each patient are reviewed separately below.

*P1. Severe Nonfluent* (*“moderate” response to C-ViC training*)

During the first session (TEST), P1 predominantly activated RH language homologues commonly activated in the LH during semantic tasks with normals. In P1, this included in part, R IFG (BA 47, 44), R MFG (BA 46, 10, 6), and R temporal areas (BA 37, 38). The RH regions with the highest levels of activation on TEST were also significantly activated on RETEST, but on RETEST, they demonstrated less spatial extent and intensity (Fig. 5 and Table 2). A notable exception to this was observed in the L temporal fusiform gyrus (TFG, BA 37) on TEST, $z = 7.1$; but on RETEST, adjacent voxels were highly activated in L TFG (BA 19), $z > 8$. Regions that were weakly activated during TEST, such as L MTG (BA 21) and R BA 44 did not reach significance during RETEST. Even though P1’s lesion spares both Broca’s and Wernicke’s cortical areas, he failed to activate either of these LH cortical regions during either TEST or RETEST.

*P2. Moderate-Severe Nonfluent* (*“best” response to C-ViC training*)

During TEST, P2 also predominantly activated RH language homologues commonly activated in the LH in normals during semantic tasks. In P2, this included in part, R IFG (BA 47), R MFG (BA 46, 10, 6), R SMA (BA 6), R TFG (BA 37), and R angular gyrus (BA 39). These significantly activated regions on TEST also were significantly activated on RETEST, although with less spatial extent and intensity. See Table 2. In spite of his large perisylvian cortical lesion, P2 also weakly activated L supramarginal gyrus (BA 40), but only during TEST. Overall, Table 2 shows that P2 had significant activation in more LH areas on TEST and/or RETEST (frontal, temporal and parietal) than P1 (frontal and temporal, only).

Table 2
Activation foci in selected Brodmann areas relevant for semantic tasks: Semantic decisions vs. rest[1]

| Area[2] | P1: TEST[3] | P1: RETEST | P2: TEST | P2: RETEST |
|---|---|---|---|---|
| *Left frontal* | | | | |
| L SFG (8) | – | – | −6,18,52 (4.9) | – |
| L SFG (6) (**=SMA) | – | – | – | −10,9,58 (5.1)** |
| L MedFG (8) | – | – | −4,28,46 (6.6) | −8,26,48 (5.2) |
| | – | – | 0,26,47 (6.5) | – |
| L MedFG (10) | – | −6,56,−6 (5.0) | – | – |
| *Left temporal* | | | | |
| L TFG (36) | −38,−38,−25 (7.5) | – | – | – |
| L TFG (37) | −44,−61,−14 (7.1) | – | −28,−51,−18 (7.3) | −42,−61,−7 (4.8) |
| | – | – | −36,−45,−13 (6.7) | −36,−63,−19 (4.6) |
| L TFG (19) | – | −46,−70,−10 (>8) | – | – |
| L MTG (21) | −61,−39,−8 (5.8) | – | – | – |
| L MTG (39) | – | – | – | −48,−60,8 (4.5) |
| L STG (39) | – | – | – | −32,−55,32 (4.1)* |
| *Left parietal* | | | | |
| L SupraMarg (40) | – | – | −42,−41,37 (3.5)* | – |
| *Right frontal* | | | | |
| R IFG (9) | 44,4,31 (7.2) | 48,7,25 (6.3) | – | 50,19,27 (4.64)* |
| R IFG (44) | 44,16,5 (4.3)* | – | – | – |
| R IFG (45) | – | – | – | 51,24,10 (3.6)* |
| R IFG (47) | 40,15,−6 (5.2) | – | 28,27,−8 (5.6) | 28,27,−6 (4.8) |
| | – | – | 38,26,−15 (4.9) | 46,18,1 (3.2)* |
| R MFG (46) | 40,34,22 (5.6) | – | 44,19,21 (7.7) | 42,18,19 (5.1) |
| | – | – | – | 50,32,9 (3.7)* |
| R MFG (10) | 38,48,−4 (6.4) | – | 38,39,13 (6.6) | 40,39,13 (4.1)* |
| | 34,46,−4 (6.3) | – | 24,54,−11 (6.4) | – |
| R MFG (6) | 30,−11,45 (>8) | 32,−11,45 (7.1) | 42,14,49 (>8) | 38,12,47 (>8) |
| | – | 42,−3,53 (7.1) | 26,3,62 (5.0) | – |
| R MedFG (6) | 8,−19,51 (6.9) | 8,−19,51 (6.3) | 2,29,35 (5.6) | – |
| R MedFG (10) | – | 4,58,−4 (5.6) | – | – |
| | – | 4,60,3 (5.0) | – | – |
| R Cing (32) | – | 6,6,47 (5.6) | – | 8,34,26 (4.3)* |
| R SFG (6) (**=SMA) | – | 24,−8,67 (5.8) | 2,16,54 (6.5)** | 6,18,54 (6.3)** |
| R MFG (9) | 44,27,26 (6.0) | 48,27,34 (5.7) | 59,21,28 (4.9) | 36,17,36 (4.2)* |
| *Right temporal* | | | | |
| R TFG (37) | 38,−59,−16 (>8) | 38,−59,−16 (7.8) | 34,−59,−11 (5.8) | 36,−51,−15 (5.6) |
| | 42,−61,−7 (7.6) | – | – | – |
| R STG (38) | 50,15,−7 (4.3)* | – | – | – |
| *Right parietal* | | | | |
| R AngG (39) | – | – | 34,−55,34 (5.5) | 32,−55,36 (4.4)* |
| | – | – | – | 34,−57,32 (4.4)* |

[1] $P < 0.001$ uncorrected; Family-Wise Error Rate $<$ 0.05 corrected ; (* = False Discovery Rate <0.05 corrected)
[2] Numbers in parentheses refer to Brodmann areas. MedFG = Medial Frontal Gyrus; TFG = Temporal Fusiform Gyrus; MTG = Middle Temporal Gyrus; MFG = Middle Frontal Gyrus; SFG = Superior Frontal Gyrus; ** = SMA; SMA = Supplementary Motor Area; SupraMarg = supramarginal gyrus; AngG = angular gyrus; Cing = Cingulate
[3] Numbers refer to stereotaxic coordinates x, y, z from Talairach & Tournoux (1988) converted from MNI space (Brett, 2003); numbers in parentheses refer to Z scores

## 4. Conclusion

This study demonstrates that fMRI can be performed with chronic, moderate-to-severe nonfluent aphasia patients and that reasonably reliable test-retest results can be obtained in this patient group. Intrasubject reliability in fMRI responses was established in two participants during identical semantic judgment tasks, with expected repetition suppression [4]. Reliability is an important finding given the enormous clinical potential for utilizing fMRI in future applications, e.g., in patient selection for appropriate treatment, monitoring of rehabilitation, and verification of treatment efficacy.

The finding of repetition suppression has also been described by Raichle and colleagues [33], and Blasi and colleagues [2] as a response decrement that is modu-

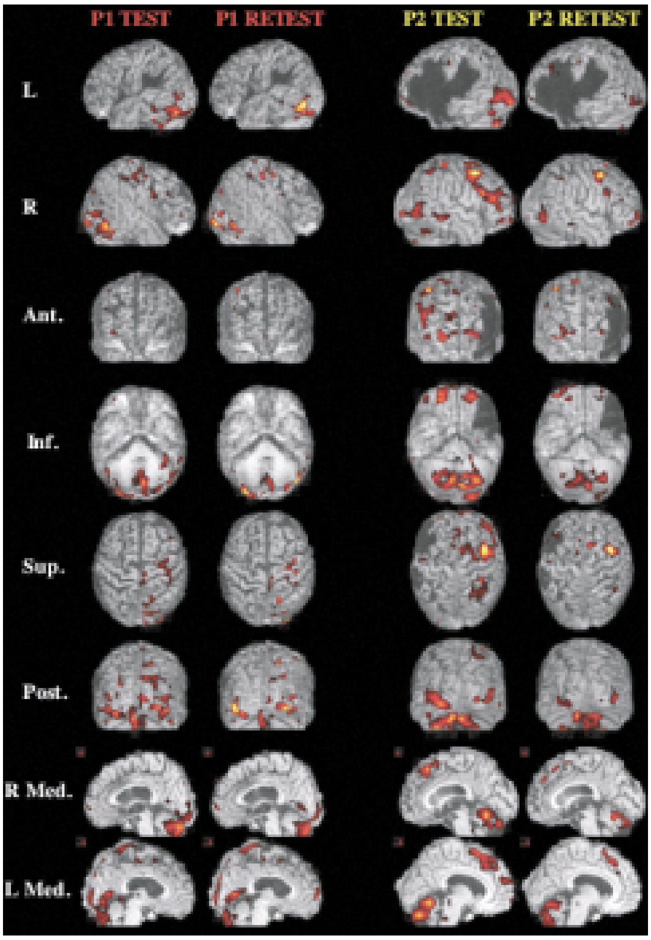

Fig. 5. Cortical activation maps for P1 (severe nonfluent) and P2 (moderate-severe nonfluent) during TEST and RETEST, superimposed on each participant's reconstructed images. Areas activated ($p < 0.001$ uncorrected, for display) during the nonverbal semantic decision task compared to a passive viewing/rest condition.

lated by practice, learning, or familiarity with the task. Raichle demonstrated decreased activations in L prefrontal cortex during a semantic task (verb generation) in normal, healthy subjects. Blasi reported similar physiological modulations of activity in R frontal cortex (dorsal IFG) in patients with L IFG damage during learning of a word stem completion task. Their results suggested that compensatory pathways in the RH may be capable of plasticity through learning.

Recently, Fridriksson and Morrow [9] examined changes in cortical activation as a result of manipulating task difficulty on a picture-word verification task. They found greater activation in the difficult condition, compared to the easy condition, for participants with aphasia and healthy, age-matched controls. Their findings, as well as those of Blasi [2], highlight the significant role of both task familiarity and task difficulty in modulating cortical activations. Their results suggest that in order to verify treatment-induced brain plasticity, these factors may need to be calibrated pre- and post-treatment.

Participants in the current study were already familiar with the task stimuli, which had comprised part of their training in C-ViC. They nonetheless both demonstrated considerable fMRI response decrements between TEST and RETEST. This decrement in both spatial extent and intensity of cortical activation was also associated with decrements in response time, although accuracy in both participants remained stable. Future investigations would benefit from using more stimuli, including some novel stimuli, as well as a mixed block/event-related paradigm, in which post-hoc analyses might reveal differences between accurate and inaccurate, easy and difficult, or fast and slow trials.

In addition to establishing intrasubject test-retest reliability in fMRI, this study also supports evidence of individual differences for LH and RH involvement in long-term aphasia recovery. P1 ("moderate response" to C-ViC training), whose predominantly subcortical lesion spares many of the LH cortical areas previously associated with semantic processing, nonetheless demonstrates a strongly RH-lateralized pattern of activation. Although his lesion does not extend into Broca's or Wernicke's cortical areas, neither of these regions (nor most of the spared LH perisylvian cortex, with the exception of L TFG and L MTG) was recruited in performance of this semantic judgment task. This failure to recruit classical cortical language areas that appear undamaged by structural MRI may be due to disconnection or diaschisis resulting from the subcortical white matter damage, or may reflect microscopic infarcts, or some combination of these factors [21,26]. As Nadeau and Crosson [26] have suggested, separating the direct effects of white matter lesions from the effects of associated vascular events (e.g., sustained cortical hypoperfusion), has not generally been possible. Future investigations utilizing converging evidence from structural and functional MRI, diffusion tensor imaging, and perfusion MRI, in conjunction with detailed behavioral data, may further address this question.

As one might expect, neither patient significantly activated identical clusters on TEST and RETEST. However, each patient activated similar parts of his own network for lexical-semantic processing on RETEST. For example, both P1 and P2 activated parts of RH homologous regions on TEST and RETEST analogous to LH regions activated on lexical-semantic tasks in normals: IFG, MFG and TFG. P2, however, the patient with "best" response to C-ViC training, was also able to activate more of these LH regions than P1, on TEST and/or RETEST, including L supramarginal gyrus.

P2 demonstrated a strongly RH-lateralized pattern of activation. Compared to P1, however, P2 showed more activation in LH frontal, temporal and parietal areas, despite large LH lesion that destroyed most of the LH cortical regions critical to semantic processing. It is tempting to characterize the differences in LH activation between our two subjects as supporting previous treatment studies. For example, new LH activation has been associated with better language outcome in aphasia patients post- speech/language therapy intervention [8,18,25,36]. Leger et al. [18], for example, studied an aphasic patient pre- and post- speech therapy on a confrontation naming (and control rhyming) task. Their patient demonstrated greater perilesional activity (including L Broca's area and the L supramarginal gyrus) as performance on the task improved. As their study suggests, it may be the case that restoration of LH language-related networks is critical for efficient or effective recovery in poststroke aphasia.

In the current study, we observed greater LH perilesional activation in the patient with more extensive long-term recovery during a nonverbal semantic judgment task. Unfortunately, one methodological weakness of our design was the lack of a true baseline task to which these two patients' pattern-viewing and semantic-processing could be compared. Not having normalized each subjects' data to this common baseline, evidence of greater LH activation must be interpreted with caution. Future investigations exploring the question of relative degrees of hemispheric recruitment in aphasia recovery should include a true resting/baseline condition.

In summary, the three hypotheses tested in the present, small study were generally supported by the results – i.e., 1) chronic aphasia patients with moderate-to-severe nonfluent speech can provide reliable fMRI data during performance of a nonverbal semantic decision task in the scanner; 2) during fMRI, our two patients activated RH regions homologous to LH regions previously associated with lexical-semantic functions in normals; and they each activated some L perilesional regions and undamaged LH regions; and 3) the patient with "best response" to C-ViC training had a different pattern of fMRI activation than the patient with "moderate response", possibly including more LH activity in the patient with "best response". Additional fMRI studies with a larger number of aphasia patients where the language tasks include a baseline condition and are calibrated to account for the modulatory effects of practice, familiarity, and task difficulty are recommended. Future investigations would also benefit from analyses which might provide converging evidence of the significance of BOLD signal activation in these patients, including lesion volume analysis, perfusion, and diffusion weighted imaging.

## Acknowledgements

Research supported by grants from the Medical Research Service, Department of Veterans Affairs, Washington, D.C., and R01 DC05672 (to M.A.N.); and P30 DC05207 to the BU Aphasia Research Center from the National Institute on Deafness and Other Communication Disorders, Bethesda, MD. The authors would like to acknowledge the assistance of the MRI staff at McLean Hospital; Ashley Young, Patricia Pimentel, Anne Smith, RT and Eileen Connolly, RT.
